# Instrumental Activities of Daily Living: The Processes Involved in and Performance of These Activities by Japanese Community-Dwelling Older Adults with Subjective Memory Complaints

**DOI:** 10.3390/ijerph16142617

**Published:** 2019-07-23

**Authors:** Yuriko Ikeda, Noriyuki Ogawa, Kazuhiro Yoshiura, Gwanghee Han, Michio Maruta, Maki Hotta, Takayuki Tabira

**Affiliations:** 1 Doctoral Program of Clinical Neuropsychiatry, Graduate School of Health Science, Kagoshima University 8-35-1, Sakuragaoka Kagoshima 890-8544, Japan; 2 Faculty of Health Science, Kyoto Tachibana University 34, Yamada-cho, Oyake Yamashina-Ku, Kyoto 607-8175, Japan; 3 Department of Neuropsychiatry, Faculty of Life Science, Kumamoto University, 1-1-1, Honjo, Chuo-ku, Kumamoto 860-8556, Japan; 4 Department of Psychiatry, Graduate School of Medicine, Osaka University 2-2 Yamadaoka, Suita, Osaka 565-0871, Japan; 5 Graduate School of Health Science, Kagoshima University, 8-35-1, Sakuragaoka Kagoshima 890-8544, Japan

**Keywords:** activities of daily living, dementia, instrumental activities of daily living, mild cognitive impairment, subjective memory complaints

## Abstract

Subjective memory complaints (SMCs) may predict the onset of dementia. The purpose of this study was to clarify characteristics of performance of activities of daily living (ADL) for older adults with SMCs and to offer support options that enable them to maintain their community-based lifestyle. A self-administered questionnaire was sent to 2000 randomly selected members of CO-OP Kagoshima, and 621 responded. 270 responders answered all questions were categorized into SMC (+) group and SMC (−) group (*n* = 133). Participants were evaluated the Process Analysis of Daily Activity for Dementia. A 2-sample t-test or the Chi-square test were used to compare the averages of continuous variables or the proportions of categorical variables. The results showed the SMC (+) group ranked significantly lower in ability to use the telephone, shop, cook, do housekeeping, manage finances, and manage medications compared with the SMC (−) group. In addition, the SMC (+) group was significantly less independent than the SMC (−) group in many processes requiring the use of tools, operation of machines, management of goods, selection of tools, and monitoring. To enable continued independence of older adults’ experiencing SMCs, it may be important to analyze their performance of ADL and to develop plans for supporting their strengths.

## 1. Introduction

In Japan, efforts to detect and prevent dementia and mild cognitive impairment (MCI) early have become increasingly urgent due to the increasing number of people with dementia in the aging society. Subjective memory complaints (SMCs) represent an awareness of memory loss and are evaluated by simple questions with a Yes/No response [1]. SMCs and changes in daily life can trigger awareness of mild cognitive decline. This is notable because it has been reported that 25% to 50% of community-dwelling older adults experience SMCs [2]. Following this report, a 3-year follow-up survey for older adults without dementia reported an increased risk of dementia (OR = 1.25, 95% CI = 1.03–1.52) when SMCs were present without objective memory loss [3]. Furthermore, a long-term follow-up study for older adults without dementia reported that SMCs could predict the onset of dementia after five years [4]. Another previous study that examined the risk of dementia based on the relevance of cognitive function and SMCs indicated that a person who was cognitively intact and experienced SMCs was approximately five times more likely to develop dementia within two years [5]. Based on the above results, SMCs may be an indicator of dementia, and rehabilitative intervention at the outset of SMCs may be effective in preventing MCI and cognitive decline.

Previous studies have shown that—even early in the disease course—individuals with MCI and mild Alzheimer’s Disease (AD) reduce their engagement in instrumental activities of daily living (IADL) [6,7,8]. A previous study examining the relationship between SMCs and well-being in older community-dwelling adults reported that SMCs were associated with low well-being, daytime sleepiness, and low IADL performance [9]. Several studies have suggested a relationship between the female gender and SMCs and IADLs [10,11]. It is conceivable that complex IADL independence is likely to decline once a person begins to experience SMCs. However, no study has thoroughly investigated the relationship between older community-dwelling adults with SMCs and their performance of daily activities. The Process Analysis of Daily Activity for Dementia (PADA-D) is an ADL assessment we developed [12]. PADA-D can divide the performance of daily living activities by those with cognitive decline into processes and analyze ADL in detail. There are some ADL scales such as Disability Assessment for Dementia (DAD) and Physical Self-Maintenance Scale (PSMS) etc. DAD evaluates ADL processes in detail by dividing them into “Start”, “Plan”, and “Execution”. However, in order to identify changes in the IADL by SMCs at an early stage, it is necessary to understand changes in process tracking in more detailed IADL items. The purpose of this study was threefold: 1) examine how CO-OP Kagoshima members perform their daily activities using PADA-D; 2) investigate whether the presence or absence of SMCs in community-dwelling adults is related to degree of independence in IADLs; 3) to consider effective forms of support that will enable older adults to continue living in their communities.

## 2. Materials and Method 

### 2.1. Study Design

This was a cross-sectional study using a self-administered questionnaire. 

### 2.2. Ethical Considerations

This study was approved by the ethics board of Kagoshima University. The questionnaire explained to participants what the purpose of the research was, and that the information collected would be kept confidential and used only for the purpose of this study. By answering the survey questions, the respondents indicated their agreement to participate. Our study protocol was approved by Ethics Committee on Epidemiological Studies, Kagoshima University on 27 December 2018. Its identification code is 170377(370)-2.

### 2.3. Participants

Consumer’s Co-operative (CO-OP) is an autonomous association of consumers who volunteer to fulfill common needs and aspirations. CO-OP Kagoshima is a private enterprise with deep ties to the local community; it assists the residents with various activities, including conducting trips to stores, delivery, and other benefits that may support functioning. At CO-OP Kagoshima, many employees in stores and delivery businesses struggle to communicate with people with dementia or suspected dementia. In December 2018, a self-administered questionnaire was sent to 2000 randomly selected members of CO-OP Kagoshima who were 60 years old and over. We received 621 responses (recovery rate: 31%). The questionnaire included an enclosed reply envelope; thus, the responses were collected vial mail. The survey period was from December 2018 to January 2019. Of the returned responses, there were 270 participants who answered the question regarding the presence or absence of SMCs and who were not missing important data. The sample size required to complete the survey was 128 people total, or 64 people for each group (effect size = 0.5, α = 0.05, power = 0.8).

### 2.4. Implementation Method

#### 2.4.1. Procedure

The participants answered a question regarding the presence or absence of SMCs after responding to a question concerning Everyday Memory Checklist (EMC) as an evaluation of SMCs. A previous study reported that direct questions about SMCs (e.g., “Have you been having memory difficulties that upset your everyday life?”) were likely to predict objective memory impairments [13]. Participants in this study were directed to answer “Yes” or “No” to the question “Do you consider yourself as being forgetful or do you ever feel inconveniences in life due to forgetfulness?” In addition, participants answered questions about personal characteristics and IADL-8 performance of PADA-D. Those who answered “Yes” about SMCs were categorized in the SMC (+) group, while those who answered “No” were categorized in the SMC (−) group.

#### 2.4.2. Measurements

##### EMC

To measure SMCs, we used EMC. EMC introduces 13 items and scenarios that may arise in real life due to memory impairment and evaluates them on a scale of 0 (not at all) to 3 (always). The sum of these 13 scores is then used to rank the level of memory impairment—the higher the score, the more significant the memory impairment. The average score of healthy older adults (between 60 and 69 years old) is 11.6 ± 5.4 [14].

##### Participant Characteristics

Participant characteristic data were collected in the questionnaire. Items assessed for age, gender, living situation (living alone or with family), subjective feelings of health (very good, good, bad, or very bad), and work status (working or not working). Other items assessed subjective pain level, subjective muscular weakness, presence or absence of visual impairment, presence or absence of hearing impairment, and hobby involvement (or hobbies).

##### PADA-D

The PADA-D is an ADL assessment that divides the performance of daily activities into processes and items. Five occupational therapists and two dementia specialists collaborated to determine the processes and items to be included from the Physical Self-Maintenance Scale and Lawton IADL scale. The PADA-D consists of a total of 14 activity performances (6 basic ADL performances and 8 IADL performances). Each activity performance is divided into five processes and three items (Table 1). As an example, Table 2 demonstrates how “Ability to use the telephone” is divided into processes and items. In PADA-D, activities performances are arranged in a time series from the beginning to the end of an action, and it is possible to clearly indicate which process is impaired. The examiner judges the item by “doing (YES) ” and “not doing (NO),” granting points for “doing (YES). ” Three points are available for 1 process, 15 points for 1 performance, and a total of 210 points for the 14 performances. The final scale with the items that were selected had high internal consistency and criterion validity (Cronbach’s α = 0.96) [15]. Previous studies showed that people with MCI experienced an early decline in IADLs. Therefore, this study investigated IADL-8 performance of PADA-D (i.e., ability to use the telephone, shop, cook, do housekeeping, use modes of transport, do laundry, manage finances, and manage medications).

### 2.5. Statistical Analysis

Once data were gathered, totaled, and averaged, participant characteristics were considered. Next, a 2-sample t-test was used to compare the averages of age. A Mann-Whitney U test was used to compare the median of EMC total score between the SMC (+) group and SMC (−) group. Next, a Chi-square test was used to compare the proportions of categorical variables (such as gender) between the two groups. Finally, we used a 2-sample t-test to compare the averages of the PADA-D total score and the IADL-8 performance score between the two groups. The two main groups were further divided into subgroups: independent (3 points) and not-independent (0–2 points) with respect to the process of PADA-D to investigate the relationship between the presence or absence of SMCs and independence of the IADL 5 process. If answers were missing, they were treated as missing values. All statistical analyses were performed using IBM SPSS Statistics ver25.0, and the significance level was set at 5% or 1%.

## 3. Results

### 3.1. EMC, Participant Characteristics

Table 3 showed the median EMC scores and participant characteristic percentages. The median EMC score was 8.0 (4.75–13.0) and the average participant age was 74.7 ± 8.2 years old. 

As a result of classifying all participants into two groups according to the presence of SMCs, there were 137 (50.7%) in the SMC (+) group and 133 (49.3%) in the SMC (−) group. The median EMC score was significantly lower in the SMC (+) group (12.0 (7.0–15.0)) than in the SMC (−) group (5 (2.5–9.0)) (*p* < 0.001). The data showed a significant association among SMCs, subjective pain, muscular weakness, difficulty seeing, and difficulty hearing. The SMC (+) group included many participants that experienced pain, muscular weakness, and difficulty seeing and hearing.

### 3.2. PADA-D IADL 8 Performance

Table 4 showed the results of the 2-sample t-test of PADA-D 8 performance for the two groups. The performance scores for ability to use the telephone (14.4 ± 2, 4, *p* = 0.013), shop (14.1 ± 3.0, *p* = 0.019), cook (13.9 ± 3.2, *p* = 0.013), do housekeeping (13.5 ± 3.3, *p* = 0.006), manage finances (12.4 ± 3.4, *p* = 0.035), and manage medications (13.9 ± 2.9, *p* = 0.028) were significantly lower in the SMC (+) group than the SMC (−) group.

### 3.3. PADA-D IADL 5 Process

**Figure 1:**
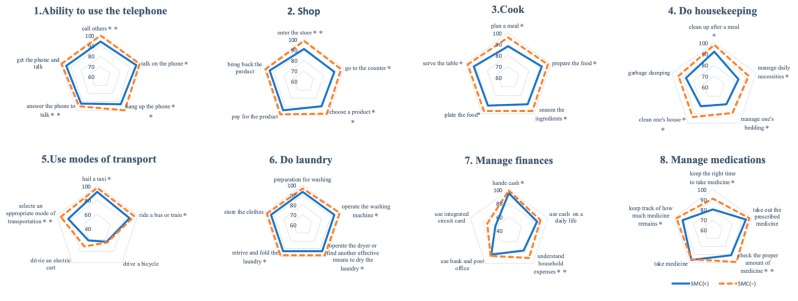
Comparison of independence of instrumental activities of daily living (IADL) process between with and without Subjective memory complaints (SMCs). The result of the Chi-square test of PADA-D 5 process for the two groups. Solid line; SMC (+) group, dotted line; SMC (−) group. * *p* < 0.05, ** *p* < 0.01.

Figure 1 showed the results of the Chi-square test of PADA-D 5 process for the two groups. 

(1) Ability to use the telephone

Independence was rated (as a percentage of independence) based on the respondents’ ability to call others (+group: 94.2%, −group: 100%, *p* < 0.001), talk on the phone (+group: 95.6%, −group: 100%, *p* = 0.015), hang up the phone (+group: 93.4%, −group: 100%, *p* = 0.003), and answer the phone to talk (+group: 94.9%, −group: 100%, *p* = 0.009). For this activity, participants in the SMC (+) group were significantly less independent than the SMC (−) group.

(2) Shop

Independence was rated based on the respondents’ ability to enter the store (+group: 91.2%, −group: 98.5%, *p* = 0.007), go to the counter (+group: 90.4%, −group: 97.7%, *p* = 0.012), and choose a product (+group: 89.0%, −group: 97.7%, *p* = 0.004). Based on the results, the SMC (+) group was significantly less independent than the SMC (−) group.

(3) Cook

Independence was rated based on the respondents’ ability to plan a meal (+group: 88.1%, −group: 96.2%, *p* = 0.016), prepare the food (wash, cut, and heat the ingredients) (+group: 91.9%, −group: 97.7%, *p* = 0.035), season the ingredients (choose seasoning, et al) (+group: 89.7%, −group: 96.9%, *p* = 0.019), plate the food (+group: 91.2%, −group: 96.9%, *p* = 0.049), and serve the table (+group: 92.6%, −group: 98.5%, *p* = 0.022). Based on the results, the SMC (+) group was significantly less independent than the SMC (−) group.

(4) Do housekeeping

Independence was rated based on the respondents’ ability to clean up after a meal (+group: 92.0%, −group: 98.5%, *p* = 0.013), manage daily necessities (+group: 83.2%, −group: 93.1%, *p* = 0.012), manage one’s bedding (+group: 78.8%, −group: 88.5%, *p* = 0.032), and clean one’s house (+group: 81.0%, −group: 93.1%, *p* = 0.003). Based on the results, the SMC (+) group was significantly less independent than the SMC (−) group.

(5) Use modes of transport

Independence was rated based on the respondents’ ability to hail a taxi (+group: 91.9%, −group: 97.6%, *p* = 0.04), ride a bus or train (+group: 88.0%, −group: 95.2%, *p* = 0.039), and select an appropriate mode of transportation (+group: 82.8%, −group: 94.1%, *p* = 0.005). Based on the results, the SMC (+) group was significantly less independent than the SMC (−) group.

(6) Do laundry

Independence was rated based on the respondents’ ability to operate the washing machine (+group: 93.4%, −group: 99.2%, *p* = 0.012), operate the dryer or find another effective means (i.e., hanging laundry) to dry the laundry (+group: 93.4%, −group: 98.5%, *p* = 0.036), and retrieve and fold the laundry (+group: 93.4%, −group: 98.5%, *p* = 0.035). Based on the results, the SMC (+) group was significantly less independent than the SMC (−) group.

(7) Manage finances

Independence was rated based on the respondents’ ability to handle cash (+group: 94.9%, −group: 99.2%, *p* = 0.035) and understand household expenses (+group: 76.5%, −group: 90.2%, *p* = 0.003). Based on the results, the SMC (+) group was significantly less independent than the SMC (−) group.

(8) Manage medications

Independence was rated based on the respondents’ ability to keep the right time to take medicine (+group: 80.3%, −group: 91.0%, *p* = 0.016), check the proper amount of medication (+group: 91.0%, −group: 99.2%, *p* = 0.003), and keep track of how much medicine remains (+group: 91.6%, −group: 97.6%, *p* = 0.038). Based on the results, the SMC (+) group was significantly less independent than the SMC (−) group.

## 4. Discussion

This study involved a cross-sectional design using a self-administered questionnaire to investigate the relationship between SMCs and IADL 8 performances of Japanese community-dwelling members of CO-OP Kagoshima. The results revealed that the SMC (+) group had a significantly lower EMC score than the SMC (−) group. The SMC (+) group had significantly lower scores for ability to use the telephone, shop, cook, do housekeeping, manage finances, and manage medications than the SMC (−) group. Furthermore, in the 27th of the 40 processes (i.e., call others, manage daily necessities, keep the right time to take medicine), the SMC (+) group was significantly less independent than the SMC (−) group.

A previous study examined the IADL of older (aged between 70 and 100 years) community-dwelling adults without dementia and suggested that a decline in activities requiring high cognitive function (e.g., doing two things at once or giving directions when asked) was a sign of MCI [16]. A longitudinal study revealed that the higher the age, the lower the self-reported IADL function, suggesting that everyday cognition could be a predictor of the self-reported IADL function [17]; another cross-sectional study involving of participants aged 60 years or older reported that women with SMCs experienced impairment in their higher-level functional capacity [10]. In addition, a prospective study for older community-dwelling people who maintained cognitive function showed that shopping and managing medications were common complaints related to their ADL difficulties [18]. Furthermore, a cross-sectional study that examined the relationship between subjective cognitive concerns (SCC) and IADL for those aged more than 50 years suggested that the higher the SCC, the lower the ability to handle finances, maintain a house, remember events, and travel independently [19]. The results of the previous studies were confirmed by the current study’s results. Nevertheless, the way in which we divided the performance of daily living activities into detailed processes and compared the presence of SMCs with the proportion of participants to conduct these processes independently was novel.

The present results showed that the SMC (+) group had significantly lower EMC scores compared to the SMC (−) group. We expected these scores to be low in the SMC (+) group because EMC is a self-administered questionnaire that assesses the frequency of problems caused by memory impairment. Comparison of PADA-D performance showed that the SMC (+) group’s ability to use the telephone, shop, cook, do housekeeping, manage finances, and manage medications were conducted at a significantly lower rate than the SMC (−) group. These actions are complex activities that require advanced cognitive functions, such as executive function, prospective memory, working memory, processing ability, and judgment. Memory, executive functions, reasoning, and processing speed decrease with age [16], and executive functions have been reported to be related to shopping, handling finances, laundry, and transportation [20]. Another previous study suggested that older adults with SMCs might experience decreased task-directed attention [21]. Moreover, diffuse white matter lesions have been linked to decreased executive function and processing speed in healthy older adults over 55 years [22], and these white matter lesions are also associated with SMCs, cognitive dysfunction, impaired basic ADL, and impaired IADL [23]. In this study, in EMC, many SMC (+) group members responded with “Sometimes there are problem behaviors”, for the questions considered to be related to short-term memory, orientation, perspective memory, executive function, and divided attention. On the other hand, most of SMC (−) group members responded “no” to those questions. In this study, we believe that IADL difficulties for the SMC (+) group were involved in short-term memory, orientation, perspective memory, executive function, and divided attention. In addition, the SMC (+) group may have influenced physical factors such as subjective pain, subjective muscular weakness, difficulty in seeing and difficulty in hearing. However, no reports compared IADL process and SMCs after dividing the performance of daily living activities into detailed processes. The findings in our study showed the SMC (+) group were significantly less independent than the SMC (−) group in the 27th out of 40 process. We thought that the 27 processes included four common elements-required to performance IADL- of using tools and operating machines, managing goods, selecting tools, and monitoring. For example, call others, prepare the food (wash, cut, and heat the ingredients), and clean one’s house contained element of using tool and operating machine. Managing daily necessities, understand household express, and keep track of how much medicine remains contained element of managing goods. Choose a product, season the ingredients (choose seasoning, et al), and select an appropriate mode of transportation contained element of selecting. Hang up the phone, enter the store, check the proper amount of medicine contained element of monitoring. In fact, data revealed that the SMC (+) group had difficulties using tools, operating machines, managing goods, selecting tools, and monitoring—activities that required memory and executive functions in various situations in daily life. 

On the other hand, there were also processes (i.e., talk on the phone, pay for the product, and garbage dumping.) in which the SMC (+) group performed to the same extent as the SMC (−) group. 

Therefore, these findings suggested that older adults with SMCs were impaired in complex IADL performance and revealed that they had difficulties in processes requiring them to use tools, operate machines, manage goods, select tools, and monitoring. Based on this, the following supports were considered necessary to enable older adults with SMCs to continue community life: (1) Dividing performance of daily living into processes and analyzing them in detail; (2) Predicting which processes will become more difficult; (3) Providing support for these processes by utilizing the abilities that the residents continue to possess. In Japan, preventive services for long- term care began in 2006 with the purpose of living independently without requiring care for the elderly as much as possible [24]. The services offer exercises, cognitive training, and so on for mild flails [25]. In the future, we believe it will be necessary to consider including the provision of programs focusing on the decline in IADL affected by SMCs. 

Several limitations of this study should be acknowledged. First, the study data were collected from a self-administered questionnaire, and there was no informant-report of engagement in these activities, and this is a source of potential bias (if possible). Second, this study did not employ objective cognitive function tests. Third, we did not use multiple regression analysis or logistic analysis because we wanted to compare IADL performance and process independence between the two groups with and without SMCs. Fourth, the participant pool was limited to members of a private enterprise. Fifth, we did not consider the influence of other environmental factors on the performance of daily living activities, nor did we consider other mental diseases common to older adults, such as depression. While the association between depression and SMCs has been suggested, cognitive decline and dementia are reported to be more deeply involved than depression [2]. In order to distinguish the impact of mental symptoms on the IADL, we would have been required to assess depression. However, in support of the previous study, this study did not evaluate depression. Finally, the participants’ education background was not investigated, and this is significant as limited education has been reported to be a risk factor for dementia. 

Despite these limitations, his study conducted original research that clarified the processes of IADL disorders and demonstrated the impacts of SMCs on these processes among older community-dwelling adults. We believe the results of the study can contribute to the creation and implementation of critical support systems for older adults with early cognitive decline.

## 5. Conclusions

Our data revealed that the presence of SMCs reduced older adults’ ability to use the telephone, shop, cook, do housekeeping, manage finances, and manage medications. In addition, the older adults with SMCs had low independence ratings in many processes requiring the use of tools, operation of machines, management of goods, selection of tools, and monitoring on IADL 8 performance. Based on these data, we believe it is necessary to analyze by specific processes how older adults with SMCs perform daily living activities in order to predict how the SMCs impair the processes and provide appropriate support to help these individuals improve or maintain these processes.

## Figures and Tables

**Table 1:** List of process of PADA-D.

IADL			BADL	
Performance	Process		Performance	Process
Ability to use the telephone	call others		Toileting	get into the restroom
	talk on the phone			sit on the toilet seat
	hang up the phone			excreting
	answer the phone to talk			do post processing
	get the phone and talk			leave the restroom
Shop	enter the store		Feeding	choose a dish
	go to the counter			hold or scoop on bite-sized food with chopsticks or spoon
	choose a product			bring food to the mouth
	pay for the product			eat food
	bring back the product			finish the meal
Cook	plan a meal		Dressing	choose clothes to wear
	prepare the food (wash, cut and heat the ingredients)			take off clothes
	season the ingredients (choose seasoning, et al)			wear clothes
	plate the food			wear and take off socks
	serve the table			wear and take off shoes
Do housekeeping	clean up after a meal		Grooming	brush teeth
	manage daily necessities			face wash
	manage one’s bedding			shave makeup
	clean one’s house			hairdress
	garbage dumping			cut nails
Use modes of transport	hail a taxi		mobility	get up
	ride a bus or train			move around
	drivie a bicycle			move through the house
	drivie an electric cart			get out of the house
	select an appropriate mode of transportation			go out to the neighborhood
Do laundry	preparation for washing		Bathing	take off clothes
	operate the washing machine			pour hot water on one’s body
	operate the dryer or find another effective means to dry the laundry			soak in the bath
	retrive and fold the laundry			wash body and hair
	store the clothes			wipe body and hair
Manage finances	handle cash			
	use cash on a daily life (purhase of food, payment of rent, et al)			
	understand household expenses			
	use bank and post office			
	use integrated circuit card			
Manage medications	keep the right time to take medicine			
	take out the prescribed medicine			
	check the proper amount of medcine			
	take medicine			
	keep track of how much medcine remains			

PADA-D; Process Analysis of Daily Activity for Dementia. IADL; Instrumental Activities of Daily Living. BADL; Basic Activities of Daily Living.

**Table 2:** Example of PADA-D (ability to use the telephone).

A Series of Processes from Calling Others to Hang Up the Phone					
Score	Process	Item	Check		Remaeks
	1. call others	(1) pick up the receiver	YES	NO	
		(2) press the call button	YES	NO	
		(3) call the number	YES	NO	
	2. talk on the phone	(1) put phone on your ear	YES	NO	
		(2) to make sure the person	YES	NO	
		(3) tell the matter	YES	NO	
	3. hang up the phone	(1) end the conversation	YES	NO	
		(2) remove the phone from your ear	YES	NO	
		(3) press the call end button	YES	NO	
	4. answer the phone to talk	(1) find the phone by noticing the ringing tone	YES	NO	
		(2) confirm the caller	YES	NO	
		(3) press the call button	YES	NO	
	5. get the phone and talk	(1) put the receiver to one’s ear	YES	NO	
		(2) to ask for a matter	YES	NO	
		(3) the response is valid	YES	NO	

Enter the score for each process in the score column after checking “YES” or “NO” for the three items.

**Table 3:** Characteristics of subjects.

Parameter	Total (*n* = 270)	SMC		*p*-Value
		(+) group (*n* = 137)	(−) group (*n* = 133)	
EMC total score, median (25th–75th percentikes)	8.0 (4.75–13.0)	12.0 (7.0–15.0)	5.0 (2.5–9.0)	0.000 ^a^
age, mean (SD), year	74.7 (8.2)	73.9 (8.6)	74.5 (7.8)	0.570 ^b^
female	245 (90.7)	128 (93.4)	117 (88.0)	0.120 ^c^
living with family	194 (71.9)	103 (75.2)	91 (68.4)	0.290 ^c^
subjective feeling of health				
very good	22 (8.1)	8 (5.8)	14 (10.5)	
good	186 (68.9)	95 (69.3)	91 (68.4)	
bad	47 (17.4)	25 (18.2)	22 (16.5)	
very bad	15 (5.6)	9 (6.6)	6 (4.5)	
working	74 (27.8)	39 (28.9)	35 (26.7)	0.690 ^c^
subjective pain	199 (74.5)	110 (80.3)	90 (68.7)	0.027 ^c^
subjective muscular weakness	122 (45.2)	76 (55.5)	45 (35.4)	0.007 ^c^
difficulty in seeing	238 (88.1)	126 (92.0)	112 (84.2)	0.049 ^c^
difficulty in hearing	196 (73.1)	112 (82.4)	84 (63.6)	0.001 ^c^
hobby	193 (72.0)	91 (67.4)	102 (76.6)	

Data are presented as n(%). ^a^ Mann-Whitney U test, ^b^ Independent t test, and ^c^ χ2 test were used. SMC; subjective memory. Complaints. EMC; Everyday Memory Checklist.

**Table 4:** Comparison on independence of IADL performance between with and without SMC.

SMC					
PADA-D IADL Performance	Total (*n* = 270)	(+) group (*n* = 137)	(−) group (*n* = 133)	*p*-Value ^a^	r
total score	107.4 (17.7)	105.5 (21.8)	109.4 (11.9)	0.066	0.13
Ability to use the telephone	14.7 (1.7)	14.4 (2.4)	14.9 (0.4)	0.013	0.21
Shop	14.4 (2.4)	14.1 (3.0)	14.8 (1.5)	0.019	0.17
Cook	14.3 (2.6)	13.9 (3.2)	14.8 (1.5)	0.013	0.18
Do housekeeping	13.9 (2.7)	13.5 (3.3)	14.4 (1.9)	0.006	0.19
Use modes of transport	10.5 (3.6)	10.7 (3.9)	10.2 (3.2)	0.266	0.07
Do laundry	14.5 (2.3)	14.2 (2.8)	14.8 (1.4)	0.065	0.13
Manage finances	12.8 (3.0)	12.4 (3.4)	13.2 (2.5)	0.035	0.13
Manage medications	14.3 (2.3)	13.9 (2.9)	14.6 (1.2)	0.028	0.17

Data are presented as mean (SD). ^a^ Independent t test was used. SMC; subjective memory complaints. PADA-D; Process Analysis of Daily Activity for Dementia. r: effect size.
